# AI-Enabled Digital Health Promotion and Prevention: Computational Literature Review

**DOI:** 10.2196/84492

**Published:** 2026-05-18

**Authors:** Mariana Girão Carrilho, Diego Costa Pinto, Rafael Wagner, Simoni F Rohden, Miguel Telo de Arriaga, Leonor Quelhas Pinto

**Affiliations:** 1 NOVA Information Management School, Universidade Nova de Lisboa, Campus de Campolide, Lisboa, 1070-312, Portugal, 351 213828610; 2 Directorate-General of Health of Portugal (Direção-Geral da Saúde), Lisboa, Portugal

**Keywords:** health promotion, artificial intelligence, AI, natural language processing, topic modeling, machine learning

## Abstract

**Background:**

Health promotion aims to strengthen individuals’ and communities’ capacity to maintain health and well-being through behavior change, empowerment, and supportive environments. Achieving this requires interventions that are timely, personalized, and scalable—qualities increasingly supported by artificial intelligence (AI). However, research on AI-enabled health promotion remains fragmented, organized primarily around technological labels rather than the intervention purposes these tools serve, limiting the cumulative understanding of how AI techniques are applied across health promotion contexts.

**Objective:**

This study systematically maps peer-reviewed research on AI-enabled digital health promotion interventions to clarify how AI techniques are organized across intervention purposes, target users, and delivery contexts.

**Methods:**

We conducted a large-scale computational literature review of 6328 peer-reviewed journal articles using natural language processing and unsupervised machine learning. Topic modeling identified latent thematic structures, and scientometric analyses examined research clusters and application patterns across health promotion contexts.

**Results:**

The analysis identified dominant application clusters organized into three broad intervention contexts: (1) AI-enabled digital technologies embedded in health promotion applications, (2) clinical and data-driven AI systems supporting preventive care and health promotion decision-making, and (3) population-level and policy-oriented applications of AI in public health promotion.

**Conclusions:**

This study provides a structured synthesis of how AI techniques are applied in digital health promotion interventions, organized by intervention context, target population, and health promotion purpose, facilitating comparison across applications beyond technological form alone. The findings support more purpose-sensitive design, evaluation, and governance of AI-enabled health promotion applications and offer a foundation for cumulative research in this rapidly expanding field.

## Introduction

Health promotion aims to strengthen individuals’ and communities’ capacity to maintain health and well-being through sustained behavior change, empowerment, and supportive environments [1-3]. It plays a central role in population health by encouraging healthier everyday behaviors and contributing to long-term well-being, while reducing avoidable pressure on health care systems [3,4]. Digital technologies have increasingly transformed how health promotion interventions are designed, delivered, and evaluated [5,6]. Within this landscape, artificial intelligence (AI) has become particularly relevant: its capacity to process large volumes of data, generate adaptive feedback, and support decision-making aligns well with the core demands of health promotion practice [7-13]. AI-enabled techniques have consequently been applied to a wide range of intervention targets, including physical activity, smoking cessation, dietary choices, preventive care, and cognitive engagement [14-18], often through mechanisms such as adaptive feedback, decision support, and gamification [19-22].

Nevertheless, the integration of AI into health promotion introduces its own set of challenges. AI is often described as an umbrella term encompassing a heterogeneous set of technologies, including chatbots, wearables, machine learning models, and natural language processing tools [8,23], each serving very different functions depending on the health promotion purpose they support. Beyond this heterogeneity, AI limitations such as interpretability and ethical concerns can undermine whether AI-enabled interventions produce transparent outcomes that are meaningful for health promotion purposes [24,25]. Health promotion interventions also differ not only in which behaviors they target but also in who they are designed for, how they are delivered, and what role AI plays in the intervention process [26,27]. Despite this complexity, existing reviews tend to focus on isolated technologies or specific domains such as workplace health [8], pain recognition [28], or emergency response [29] and are typically organized around technological labels rather than the health promotion functions these tools serve [26,30-34]. For researchers, this makes it difficult to build cumulatively on prior work; for practitioners and policymakers, it offers little guidance on which AI approaches are appropriate for which intervention contexts. As a result, the field lacks a coherent map of how AI techniques are applied across intervention purposes, target populations, and delivery contexts, and of what a purposeful, evidence-based integration of AI into health promotion actually looks like.

To address these gaps, this study conducts a large-scale computational review of peer-reviewed research on AI-enabled digital health promotion interventions. Using natural language processing and unsupervised machine learning, we systematically map how AI techniques are applied across health promotion contexts to produce a structured analysis that clarifies what AI is being used for, by whom, and to what end. In doing so, the study makes 3 contributions. First, it conceptualizes AI in functional roles within interventions, rather than technological categories, providing a clearer basis for comparative analysis. Second, it articulates a multilevel intervention architecture integrating individual-, organizational-, and population-level applications within a single conceptual perspective. Third, it clarifies implications for evaluation, showing that effectiveness, validity, and potential harm are inseparable from the intervention purpose AI techniques serve, highlighting the need for purpose-sensitive evaluation strategies in applied digital health research. Together, these contributions are intended to support researchers, practitioners, and policymakers in navigating a rapidly growing but fragmented evidence base, and in designing future interventions where AI capabilities are matched to health promotion goals.

## Methods

### Data Collection

To systematically synthesize research on AI-enabled digital health promotion, we conducted a large-scale computational literature review combining topic modeling and scientometric analysis [23,35,36]. This dual approach is well-suited to digital health research, as it allows the identification of dominant research themes while simultaneously revealing how they are structured, connected, and developed across disciplines.

Topic modeling was used to identify latent thematic patterns in the literature, capturing how AI-enabled digital technologies are discussed and applied within health promotion research. Scientometric techniques, including coauthorship, co-word, and cocitation analyses, were used to contextualize these themes within the broader intellectual structure of the field. Together, these methods provided a robust foundation for identifying research topics relevant to health promotion practice and for synthesizing dispersed evidence across digital health, public health, and AI research.

Importantly, the analytical strategy was guided by an intervention-function perspective. Rather than treating AI technologies as the primary unit of analysis, the review focused on the functions AI-enabled systems serve within health promotion interventions: how they are embedded across delivery contexts, target different user groups, and support distinct intervention purposes. This perspective informed both the interpretation of emergent topics and their aggregation into higher-level intervention contexts, ensuring that the resulting work reflects the health promotion goals these technologies are meant to serve instead of their technical properties.

To identify relevant studies, we used a comprehensive search strategy targeting the intersection of the terms *artificial intelligence*, *healthcare*, and *health promotion*, using the Boolean operator “AND.” To ensure the inclusion of validated scholarly contributions, we applied the *ar* filter to restrict the sample to peer-reviewed journal articles, excluding conference papers and other periodicals [23,37].

**Figure 1.**
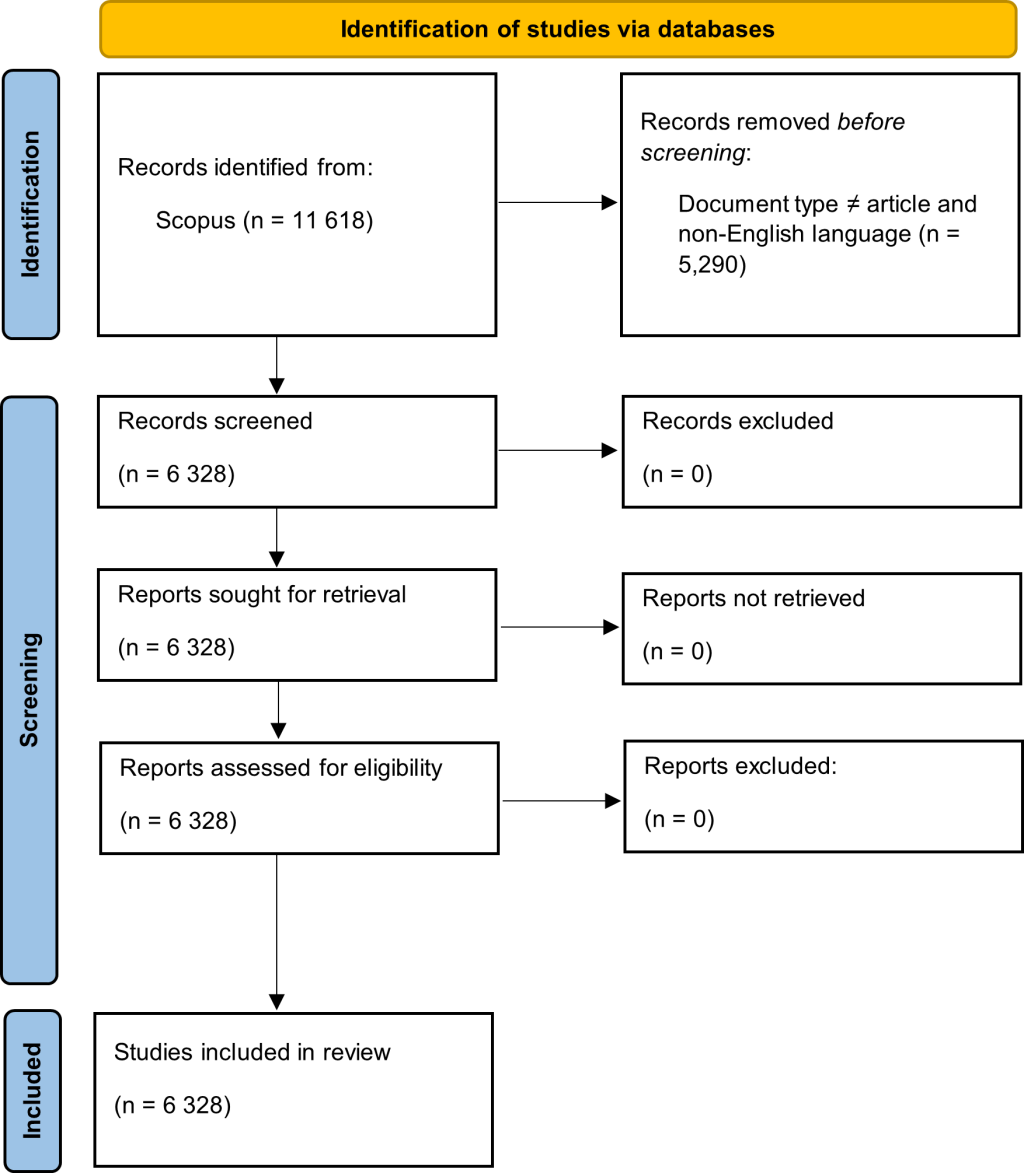
PRISMA flowchart.

The search was limited to English-language articles, which is standard practice in computational text analysis and topic modeling due to the need for linguistic consistency across documents [38]. Including multiple languages could introduce semantic heterogeneity, compromising topic coherence and interpretability. The final search query was as follows: (ALL (artificial intelligence) AND ALL (healthcare) AND ALL (health promotion)) AND (LIMIT-TO (DOCTYPE , "ar")) AND (LIMIT-TO (LANGUAGE , "English")) (Figure 1).

We selected Scopus as the primary database, as it is one of the largest and most widely used abstract and citation databases, with broad multidisciplinary coverage [39,40]. Its scope allowed the inclusion of research from digital health, medicine, public health, and related fields, making it a well-established source for systematic reviews and bibliometric analyses [41].

The search yielded 6328 articles. To further ensure methodological appropriateness, the analytical approach was discussed with 3 academic experts and 2 health management professionals, who confirmed its suitability for the study’s objectives. Consistent with capturing the breadth of AI-enabled digital health promotion research, the strategy intentionally favored inclusiveness over restrictive filtering, acknowledging that health promotion interventions often span multiple disciplines and application contexts.

### Text Preprocessing

Before topic modeling, all article abstracts were systematically preprocessed to reduce noise, ensure linguistic consistency, and enhance semantic coherence. Preprocessing was implemented in Python (Python Software Foundation) using standard natural language processing libraries, including *pandas* for data handling, *re* for regular expressions, and the Natural Language Toolkit (NLTK) for tokenization, stop-word removal, and lemmatization.

All abstracts were first converted to lowercase to enable case-insensitive matching. Noninformative elements, such as URLs, web links, email addresses, and user mentions, were removed using regular expressions. Punctuation and nonalphanumeric characters were then removed to reduce sparsity and prevent the assignment of topic weight to syntactically irrelevant tokens.

The cleaned text was subsequently tokenized into individual words using NLTK’s *word_tokenize* function. Common English stop words (eg, “and,” “the,” and “of”) were removed using NLTK’s predefined stop-word list, as these high-frequency terms contribute little to semantic differentiation across topics. The remaining tokens were then lemmatized using the WordNet lemmatizer to reduce inflected forms to their base lexical representation (eg, “interventions,” “intervening,” and “intervened” are reduced to “intervention”). This step preserved semantic interpretability while reducing dimensionality. The resulting lemmatized tokens were recombined to form the cleaned textual input for topic modeling.

### Topic Modeling and Latent Dirichlet Allocation

Latent Dirichlet allocation was applied to the preprocessed abstracts to identify latent thematic structures within the literature. Latent Dirichlet allocation is a widely used unsupervised machine learning technique that models each document as a probabilistic mixture of topics, with each topic represented as a distribution over words [36]. The method is particularly appropriate for large-scale literature synthesis, as it allows themes to emerge inductively without imposing predefined categories.

Model estimation was performed using Gibbs sampling, which iteratively updates topic assignments by sampling from the conditional probability of topics given observed words. Two hyperparameters govern the model: alpha (α), which controls the distribution of topics within documents, and beta (β), which controls the distribution of words within topics. Higher values of α allow documents to contain a broader mix of issues, whereas higher values of β permit topics to be characterized by a broader range of terms.

Consistent with standard practice in exploratory topic modeling and computational literature reviews, symmetric Dirichlet priors were specified for both the document-topic (α) and topic-word (β) distributions [23,36,42]. According to the scikit-learn documentation, symmetric priors are appropriate when the objective is to uncover general thematic structures across heterogeneous corpora rather than to impose assumptions about topic sparsity or dominance [43].

To assess robustness, we conducted a sensitivity check by comparing models estimated with symmetric and asymmetric α priors while holding other parameters constant. Across specifications, topic structures, dominant keywords, and coherence scores were highly stable, with no substantive differences in interpretation or clustering. In line with these results and prior methodological recommendations, we retained the symmetric prior specification in the final model [23,36].

Following prior computational reviews, we prioritized topic coherence and interpretability over extensive hyperparameter optimization, as the primary aim was to map the field’s intellectual and intervention-relevant structure [23]. The optimal solution consisted of 8 topics, and coherence diagnostics (coherence score=0.4956) indicated that it balanced interpretability and thematic differentiation, making it suitable for synthesizing a large and heterogeneous corpus (Multimedia Appendix 1).

Topic interpretation proceeded in 3 stages. First, we examined the terms with the highest weights within each topic. Second, descriptive labels were assigned based on the semantic content and contextual use of these terms within the articles. Third, the identified topics were evaluated against the study’s intervention-function perspective to ensure their relevance to digital health promotion. These 8 topics formed the basis for identifying higher-level groupings, which were subsequently aggregated into 3 intervention contexts: embedded, clinical, and population-level applications of AI-enabled digital health promotion.

### Ethical Considerations

This project was approved by the institutional ethics committee (project number DDMKT2026-1-203145).

## Results

### Intertopic Relationships and Emergent Clustering Patterns

The topics that structure the academic literature on AI-enabled health promotion, in order of prevalence, are societal and policy implications of digital health technologies, Internet of Things (IoT) and AI, behavioral responses to health communication, AI in clinical research, digital health interventions, AI-driven diagnosis, health apps and games, and wearable technologies. The details of each topic are presented in the Multimedia Appendix 1. To examine how the identified topics relate to one another across the corpus, we analyzed the distribution of articles using t-distributed stochastic neighbor embedding (t-SNE). This technique projects complex high-dimensional information into a 2D map so that relationships between documents can be visually inspected. Consumer-facing research, such as studies on mobile health apps, games, and interactive systems, occupies a central and overlapping region of the map, reflecting the frequent integration of behavioral and technological design concerns. Wearable technologies and IoT applications form more compact, specialized clusters, while research on societal, ethical, and policy implications is more diffusely distributed, suggesting that governance and trust considerations saturate multiple research streams rather than constituting a stand-alone domain. Clinical and data-driven research forms a more delineated cluster, centered on analytical methods and preventive decision support rather than direct intervention delivery. These patterns are corroborated by the topic-term analysis, where crosscutting concepts such as users, design, and digital systems suggest convergence between front-end intervention delivery and back-end analytical infrastructures.

**Figure 2.**
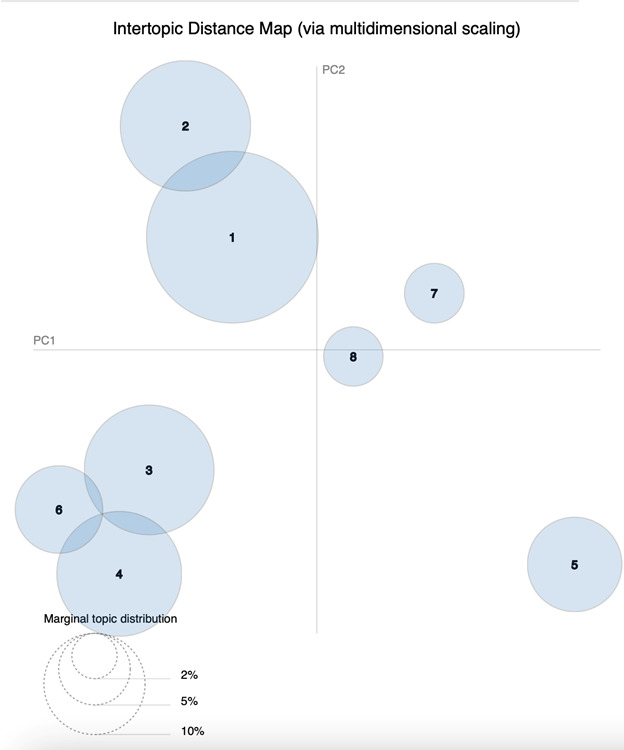
Intertopic distance map based on multidimensional scaling (MDS): circles represent the 8 identified topics, with circle size corresponding to the relative prevalence of each topic in the corpus. Distances between circles reflect semantic dissimilarity between topics, projected onto the first 2 principal components (PC1 and PC2) derived from the MDS analysis.

To further examine relationships among the 8 identified topics, we analyzed their relative positions using intertopic distance mapping based on multidimensional scaling (MDS). This technique allows complex similarity relationships between topics to be represented in a 2D space that can be visually interpreted. In the resulting map (Figure 2), each circle represents a topic, and the distance between circles reflects how similar or different the topics are based on their word distributions. Topics positioned closer together share more similar vocabularies and thematic content, whereas topics located farther apart are more distinct. The axes labeled PC1 and PC2 correspond to the first 2 principal components derived from the dimensionality reduction procedure and serve as visualization coordinates, summarizing the main variation in the distances between topics so that these relationships can be displayed in 2 dimensions.

The intertopic distance map shows that topics 1 (health apps and games) and 2 (digital health interventions) are close together and account for a substantial proportion of the corpus. Their spatial proximity reflects strong thematic overlap, suggesting that much of the literature integrates behavioral intervention objectives with considerations of digital design, usability, and engagement. These topics occupy a central position across both visualizations, indicating their foundational role in AI-enabled health promotion research.

Topic 3 (wearable technologies) appears adjacent to this central cluster but forms a more compact grouping. Its relative positioning suggests conceptual alignment with consumer-facing interventions while maintaining a distinct emphasis on embodied, sensor-based monitoring and continuous data collection. This pattern indicates that wearables function as a specialized but integral component of everyday digital health promotion interventions.

In contrast, topics 4 (AI in clinical research) and 5 (AI-driven diagnosis) are positioned further from the central consumer-facing cluster. Topic 4 forms a delineated grouping associated with methodological development, clinical data analysis, and research infrastructure, while topic 5 appears more isolated, reflecting its narrow focus on diagnostic accuracy and algorithmic performance. Their distance from topics 1 to 3 indicates that, although these streams contribute to health promotion indirectly, they are less tightly coupled with behavioral intervention delivery and user engagement.

Topics 6 (societal and policy implications of AI), 7 (behavioral responses to health communication), and 8 (IoT and AI) occupy positions that bridge multiple research streams. Topic 6 is distributed broadly across the intertopic space, underscoring its role as a crosscutting theme that intersects with ethical, governance, and trust-related concerns across contexts. Topic 7 is positioned near both consumer-facing and population-oriented research, reflecting its focus on how individuals and groups respond to digitally mediated health communication. Topic 8 occupies a nearby but distinct position, linking infrastructure-oriented research with large-scale deployment and system integration.

Taken together, the t-SNE and MDS analyses indicate that the literature organizes into higher-level groupings defined not by specific AI techniques but by how digital technologies are embedded within health promotion activities. These clustering patterns suggest the emergence of broader intervention contexts that reflect differences in delivery setting, target users, and intervention purpose. This emergent structure provides the empirical basis for the intervention-context synthesis development presented in the following section.

### The AI-Enabled Health Promotion Intervention Map

Integrating insights from the topic modeling, t-SNE visualization, and intertopic distance analysis, the 8 identified topics can be meaningfully organized into 3 broader intervention contexts that structure the systematic mapping of AI-enabled health promotion intervention (Table 1). Interventions may vary in delivery modality, ranging from unimodal systems that rely on a single modality, such as text or voice, to multimodal systems that combine multiple elements. They also differ in degree of embodiment, with some delivered through embodied technologies, such as apps, wearables, and sensor-based devices, and others implemented through more disembodied systems embedded in digital platforms, dashboards, or large-scale information infrastructures. In addition, interventions vary in their target populations, addressing individual users such as consumers and patients; institutions and professionals such as clinicians, health care providers, and researchers; or populations at scale, including policymakers and public health actors. Finally, AI-enabled interventions differ in their roles, ranging from personalization and support to decision support and large-scale coordination.

**Table 1.** Summary of how artificial intelligence (AI) techniques are applied across health promotion contexts.

Dimension	Cluster 1: embedded digital health promotion technologies	Cluster 2: clinical and data-driven health promotion technologies	Cluster 3: population-level, societal, and policy-oriented technologies
Associated topics	Topics 1 (health apps and games), 2 (digital health interventions), and 3 (wearable technologies)	Topics 4 (AI in clinical research) and 5 (AI-driven diagnosis)	Topics 6 (societal and policy implications of AI), 7 (behavioral responses to health communication), and 8 (IoT^a^ and AI)
Modality	Primarily multimodal (text, audio, visual, and/or haptic)	Primarily unimodal (analytical), with some multimodal elements (dashboards)	Primarily unimodal
Degree of embodiment	Often embodied (wearables, mobile devices, IoT, and consumer interfaces)	Mostly disembodied (back-end systems, analytical platforms, dashboards, and algorithms)	Disembodied (institutional platforms and public information systems)
Role of AI within the intervention	Monitoring, self-tracking, personalized and adaptive feedback, real-time responsiveness, motivation, and behavior change support	Risk stratification, prediction, optimization, and decision augmentation	Surveillance, population risk identification, health communication, and policy support
Intervention context	Everyday life and routine health behaviors	Clinical, organizational, and preventive care settings	Public health systems, population communication, and governance
Target population	Individuals (consumers and patients)	Professionals and organizations (clinicians, health care providers, hospitals, and researchers)	Institutions, populations, and policymakers
Primary health promotion purpose	Sustaining engagement and supporting individual behavior change and well-being	Supporting preventive decision-making and health promotion planning	Improving population health, equity, trust, and system-level coordination

a IoT: Internet of Things.

The first intervention context, embedded digital health promotion technologies, primarily comprises topics centered on consumer-facing and everyday digital interventions. This includes topic 1 (health apps and games) and topic 2 (digital health interventions), which occupy central and overlapping positions in the t-SNE and MDS maps. Their proximity reflects a strong integration of behavioral intervention goals with technological design, usability, and engagement considerations. Topic 3 (wearable technologies) also aligns with this context, forming a more compact but adjacent cluster that emphasizes sensor-based monitoring and embodied interaction. Together, these topics represent research focused on AI-enabled interventions embedded in daily routines to support behavior change, self-monitoring, and sustained engagement at the individual level.

The second intervention context, clinical and data-driven health promotion technologies, is anchored in topics 4 (AI in clinical research) and 5 (AI-driven diagnosis). These topics appear more delineated and peripheral relative to consumer-facing intervention topics, reflecting a distinct research stream focused on analytical performance, methodological development, and decision support rather than direct behavioral intervention delivery. Topic 4 emphasizes the use of AI for data analysis, research infrastructure, and knowledge generation, whereas topic 5 focuses more narrowly on diagnostic accuracy and algorithmic performance. Their relative isolation in the intertopic maps indicates that, although relevant to health promotion, these topics primarily operate within institutional and professional contexts. As such, the intervention’s purpose is mainly to support preventive decision-making and health promotion planning.

The third intervention context, population-level, societal, and policy-oriented digital health promotion, is primarily reflected in topic 6 (societal and policy implications of AI) and topic 7 (behavioral responses to health communication), with topic 8 (IoT and AI) occupying a nearby but distinct position. Topic 6 is widely distributed across the t-SNE map, underscoring its role as a crosscutting theme that intersects with multiple research streams through concerns related to ethics, governance, trust, and equity. Topic 7 focuses on how populations respond to digitally mediated health communication, linking AI-enabled systems to public engagement, persuasion, and behavioral outcomes at scale. Topic 8 bridges infrastructure-oriented research with population-level applications, reflecting the role of interconnected systems and platforms in enabling large-scale health promotion initiatives. Overall, the intervention context primarily refers to disembodied technologies, including institutional platforms and public information systems, in which AI is commonly focused on aspects such as population risk identification, health communication, and policy support.

## Discussion

### Principal Findings

This study examined the rapidly expanding literature on AI-enabled health promotion through a computational lens, mapping how research in this domain is organized across intervention purposes, target users, and delivery contexts. Our findings show that AI functions not as a stand-alone intervention but as an enabling layer that amplifies core health promotion capacities, including personalization, feedback, prediction, and coordination [5,26], within broader digital health systems.

This has important consequences for both what AI can contribute to health promotion and the challenges its integration introduces. Framing AI as an enabling layer helps explain why similar technologies produce different outcomes across contexts. A chatbot, for instance, may effectively support smoking cessation when it delivers personalized, timely feedback aligned with a user’s behavioral goals, yet prove ineffective when deployed without clear intervention logic or user fit. What drives effectiveness is therefore not the technology itself but whether its capabilities are meaningfully integrated into the intervention, suited to the target users, and oriented toward a specific health promotion purpose [44,45]. The topic modeling results support this interpretation. Rather than forming independent research streams organized around AI techniques, the corpus systematically links AI-related terms to intervention functions (eg, engagement support, self-monitoring, decision support, and large-scale coordination), suggesting that AI is consistently discussed in relation to the role it plays within an intervention, not in isolation.

At the same time, this heterogeneity creates a challenge. Because AI is embedded across such diverse intervention contexts and purposes, from individual behavior change apps to clinical decision support to population surveillance, it becomes difficult to compare findings across studies, agree on what counts as effectiveness, or build knowledge cumulatively. Without a shared way of describing what AI is doing within an intervention, evidence remains fragmented and evaluation standards inconsistent.

The analysis also reveals that this heterogeneity is organized by contextual intervention logics rather than by computational methods. Topics cluster around everyday or embedded interventions, clinical and organizational applications, and population-level or policy contexts, reflecting systematic differences in target users, delivery environments, and public health functions [46,47]. User-facing apps, games, and digital health interventions form a central and overlapping cluster; wearable technologies constitute a more specialized adjacent stream; clinical research and diagnostic applications appear more clearly delineated; and societal and policy-oriented themes cut across clusters rather than forming a single technological domain. Taken together, these patterns suggest that what unifies AI-enabled health promotion research is not a shared computational technique but a shared intervention context and purpose, a finding that directly motivates the multilevel architecture and evaluation implications developed in the sections that follow.

### Contributions to Digital Health Research

This study advances the literature in 3 ways. First, framing AI as a component of intervention purpose rather than a stand-alone technology complements existing research organized around technological labels such as chatbots, wearables, or large language models [5,26,45]. While useful for documenting capabilities, technology-centric taxonomies offer limited insight into why similar tools yield different outcomes across settings [48]. An intervention-function perspective addresses this by grounding comparative analysis in mechanism and context rather than technical form.

Second, the findings reveal a latent multilevel intervention architecture integrating individual, organizational, and population-level health promotion within a single conceptual space. Prior research has typically examined these levels in isolation [30,49,50]. Our findings suggest they are better understood as interconnected components of a broader health promotion ecosystem, structured by differences in who acts, at what scale, and under what governance conditions. Third, the findings contribute to ongoing debates about evaluation in digital health. Standard approaches, particularly randomized controlled trials and short-term behavioral outcomes, are poorly suited to assessing AI-enabled systems whose effects are diffuse, infrastructural, or emergent over time [51,52]. Evaluation criteria should be aligned with the intervention context and purpose AI supports, rather than applied uniformly across fundamentally different applications.

### Practical and Public Policy Implications

These findings have direct implications for researchers, practitioners, and policymakers. For researchers, the results highlight the need to shift from technology-centric comparisons toward function-driven study designs that specify the intervention purpose AI is expected to support, how that function operates within a given context, and the mechanisms linking AI capabilities to health promotion outcomes. For practitioners, the key question is not whether an intervention is “AI powered” but whether its capabilities align with the intended users, delivery environment, and health promotion goals, recognizing that behavior change applications demand personalization and feedback; clinical settings require decision support and transparency; and population-level initiatives demand scalability and trust. For policymakers, the findings reinforce the idea that AI-enabled health promotion raises context-specific ethical and regulatory challenges that cannot be addressed through uniform governance standards, including autonomy and privacy at the individual level, professional oversight at the organizational level, and equity and public trust at the population level.

### Limitations and Future Directions

This study has several limitations that should be considered when interpreting the findings. First, the computational analysis relied on abstracts of peer-reviewed journal articles indexed in the Scopus database and published in English. While this approach ensured methodological consistency and scholarly quality, it may have excluded relevant work published in other languages, conference proceedings, or practitioner-oriented outlets, particularly in rapidly evolving areas of digital health.

Second, the use of topic modeling and dimensionality reduction techniques necessarily involves methodological choices that influence how themes are represented and grouped. Although robustness checks indicated stable topic structures, the identified topics and clusters reflect probabilistic patterns in the data rather than definitive categorizations of the field. As such, the findings should be viewed as a guiding structure rather than an exhaustive or fixed taxonomy.

These limitations point to promising directions for future research. One important avenue is to extend computational mappings to full-text analyses, implementation reports, or mixed corpora that include policy documents and practitioner guidelines. Such extensions would enable finer-grained analysis of intervention mechanisms, deployment strategies, and real-world constraints. Longitudinal analyses could further examine how AI-enabled health promotion research evolves, revealing whether intervention contexts converge, hybridize, or diverge as technologies mature and regulatory environments change.

### Conclusion

This study offers a structured synthesis of a rapidly expanding and fragmented literature by reframing AI-enabled health promotion through an intervention-function lens. As AI becomes increasingly integrated into health promotion efforts, the central challenge for researchers, practitioners, and policymakers will not be whether to adopt AI but how to design, evaluate, and govern AI-enabled interventions in ways that meaningfully advance health and well-being.

## Supplementary material

10.2196/84492 Multimedia Appendix 1 Descriptions of topics and topic coherence diagnostics across numbers of topics.

10.2196/84492 Checklist 1 PRISMA checklist.

## Abbreviations

AI: artificial intelligence
IoT: Internet of Things
MDS: multidimensional scaling
NLTK: Natural Language Toolkit
t-SNE: t-distributed stochastic neighbor embedding
