# Supplementary material for: AI-Enabled Digital Health Promotion and Prevention: Computational Literature Review
Source: JMIR AI. 2026 May 18;5:e84492. doi: 10.2196/84492 (PMC13182876; doi:10.2196/84492)
Supplement: Multimedia Appendix 1 [file ai-v5-e84492-s001.docx]

Appendix

| *#* | *Name* | *Description* | *Sample Keywords* |
| --- | --- | --- | --- |
| 1 | Health apps and games | This topic (6.49% \| n = 411) emphasizes the design and development of digital health tools, such as apps and games, with a focus on usability and interactivity, to promote health behaviors. | user, design, application, mobile, behavior, gamification, feedback, usability, framework, metaverse. |
| 2 | Digital health interventions | This topic (9.88% \| n = 625) is focused on interventions through apps and digital tools to improve physical and mental health outcomes, especially for specific populations like older adults, children, and those with chronic conditions. | health, intervention, physical, activity, app, behavior, mental, usability, feasibility, weight. |
| 3 | Wearable technologies | This topic (3.63% \| n = 230) highlights the use of wearable devices and virtual reality (VR) environments for monitoring stress, physical activity, and safety, with applications in urban and workplace health. | wearable, virtual reality, stress, sensor, environment, safety, urban, monitoring, rehabilitation, well-being. |
| 4 | AI in clinical research | This topic (13.24% \| n = 838) centers on AI applications in medical education, training, and clinical research to improve healthcare delivery and knowledge dissemination. | study, research, education, medical, clinical, training, ChatGPT, systematic, database, learning. |
| 5 | AI-driven diagnosis | This topic (8.64% \| n = 547) explores machine learning models and algorithms for predicting diseases, assessing risks, and improving early detection and treatment of health conditions. | patient, model, disease, risk, prediction, algorithm, treatment, accuracy, diagnosis, machine. |
| 6 | Societal and policy implications of digital health technologies | This topic (23.91% \| n = 1513) focuses on the societal and policy implications of digital health technologies, including sustainability, ethical challenges, and access to healthcare. | healthcare, digital, policy, innovation, sustainability, ethical, adoption, stakeholder, economic, public. |
| 7 | Behavioral responses to health communication | This topic (16.77% \| n = 1061) analyzes public behavior and perception in response to health information and campaigns, particularly during the COVID-19 pandemic. | social, health, behavior, COVID-19, intention, public, perception, trust, vaccination, psychological. |
| 8 | IoT and AI | This topic (17.43% \| n = 1103) focuses on integrating IoT and AI technologies in healthcare, addressing security, efficiency, and intelligent systems challenges. | IoT, algorithm, network, security, privacy, healthcare, blockchain, intelligent, edge, monitoring. |

Table 1. Description of the topics.


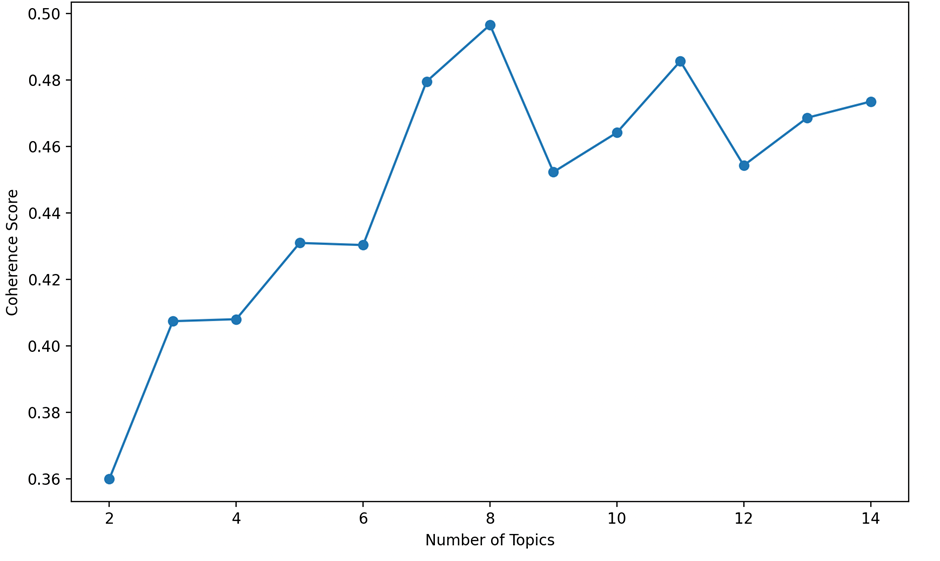


Figure 1. Topic coherence diagnostics across different numbers of topics: The figure shows coherence scores for topic models estimated with varying numbers of topics. Higher coherence values indicate greater semantic consistency within topics. The selected number of topics balances interpretability and coherence, as indicated by the plateau in coherence scores.
